# Biomechanical testing of distal femur osteotomy plate fixation techniques: the role of simulated physiological loading

**DOI:** 10.1186/s40634-014-0001-1

**Published:** 2014-06-26

**Authors:** Justus-Martijn Brinkman, Christof Hurschler, Jens Agneskirchner, Philip Lobenhoffer, René M Castelein, Ronald J van Heerwaarden

**Affiliations:** Department of Orthopaedics, Limb deformity reconstruction unit, Sint Maartensclinic, Woerden, The Netherlands; Labor für Biomechanik und Biomaterialien, Orthopädische Klinik der Medizinischen Hochschule Hanover, Hanover, Germany; Sportsclinic Germany, Hanover, Germany; Department of Orthopedics, University Medical Centre Utrecht, Utrecht, The Netherlands

**Keywords:** Plate fixation, Biomechanical testing, Femur osteotomy, Fracture, Stability

## Abstract

**Background:**

Implants for fracture and/or osteotomy fixation are often tested according to basic mechanical test models such as open gap tests or 4-point-bending tests. These may be suitable to test and compare different implants for safety and clinical approval, but are not always representative of the post-operative situation, which is decisive when it comes to bone healing. In the current study the Knee Expert Group of the Association for the Study of Internal Fixation has compared the available open gap test results of the latest version of the TomoFix Medial Distal Femoral Plate and the antecedent plate design, with the test results of a more physiological and life-like test model. In the open gap test model the antecedent plate design was found to have superior stiffness and fatigue strength.

**Methods:**

In the current study simulated postoperative conditions for medial closing wedge supracondylar osteotomies were used. The constructs were subjected to cyclical axial and torsional loading and were subsequently tested to failure.

**Results:**

The more life-like tests in this study showed that the latest version was either more or equally stable and stiff than the antecedent version of the plate, in all of the tests. It is argued that the difference in results between the two loading models is due to differences in test design.

**Conclusions:**

These test results stress the importance of not only using standard open gap and 4-point-bending tests, but also to use as life-like as possible test conditions for any form of biomechanical testing of new implants.

## Background

Implants for fixation of fractures and osteotomies have since long been tested for safety and clinical approval and have been compared to each other in so called open gap or 4-point-bending biomechanical test models [1–6]. The biomechanical loading protocols used aim at testing construct stability and fixation strength to bone and include in general axial and torsion loading, stiffness and fatigue testing. Although the open gap model purposely represents a worst case scenario of implant loading in unstable fractures, for many fractures and closing wedge osteotomies however this model represents a non-physiological test situation. The reduced fracture fragments or closed osteotomy gap are likely to improve the overall stability and stiffness of the construct [7–9].

In recent years so called Locking Compression Plates (LCP) have been developed specifically for fixation of osteotomies around the knee. Various factors have been identified to influence construct stability of LCP in plate fixation techniques; working length (i.e. distance of the first screw to the fracture), number of screws, distance of the plate to the bone, gap size and plate length [2]. Increased working length, fewer screws, a greater distance of the plate to the bone, an increased gap size and a shorter plate all negatively influenced the stability of the construct [2]. These parameters have been investigated using composite cylinders and finite element analysis (FEA) under simulated non-physiological loading conditions [2].

**Figure 1:**
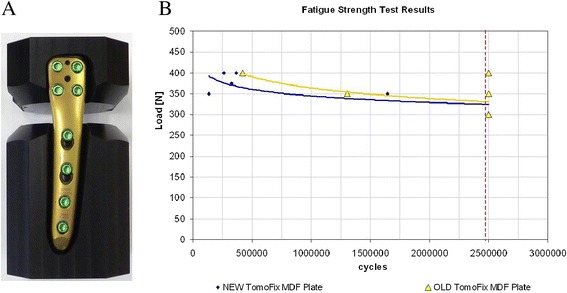
**Open gap test setup and results. A.** Anatomical MDF plate fixated in composite cylinder blocks for open gap model testing. **B.** Condensed schematic representation of the open gap fatigue tests as provided by the manufacturer (Synthes GmbH). Both plates were setup to be tested over 2500000 cycles (vertical line = end point of test runs), 2 of 5 antecedent MDF plate design plates (triangle symbol) and 5 of 5 anatomical MDF plate design plates (diamond symbol) failed before reaching the tests end at the various loading levels used (300, 350 and 400 N, antecedent MDF plate and 350,375 and 400 N, anatomical MDF plate plate). Trend lines for both are also shown (bottom line = anatomical MDF plate, top line = antecedent MDF plate).

The latest version of the TomoFix Medial Distal Femoral Plate (MDF) (Synthes GmbH, Switzerland), has shown equal static strength but inferior stiffness and fatigue strength when compared to the antecedent plate design under these non-physiological testing conditions (Figure 1A and B), performed in house by the plates manufacturer.

These results were evaluated by a group of osteotomy experts involved in the development of plates for fixation of osteotomies around the knee; the Knee Expert Group (KNEG) of the Association for the Study of Internal Fixation (AO/ASIF). They questioned the validity and the clinical relevance of the open gap model in the testing of closing wedge osteotomy techniques. It was argued that open gap and 4-point-bending tests, while being necessary from a safety standpoint, test the material and design properties of the implant, but are not a representation of the stability of the whole construct, once it is implanted in the patient.

**Figure 2:**
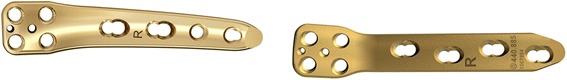
**Both the anatomical MDF plate, left, and the antecedent MDF plate, right.** The anatomical MDF plate has an optimized shape, to better fit the distal femur; it has improved screw-hole directions, and is shorter and slimmer, but equally thick.

A validated biomechanical testing model with life-like simulated loading conditions has been previously used to compare the construct stability of different types of plate fixation techniques for distal femur osteotomies, one of which was the antecedent version of the MDF plate [7,8]. The new design MDF plate has a different shape than its predecessor; it has a more anatomical pre-formed shape resulting in an improved fit to the distal femur which reduces the distance of the plate to the bone, subsequently reducing leverage forces resulting on the plate (Figure 2). It has an optimized screw-hole orientation, providing a better fit for the screws in the femur. It has also been made slightly shorter and slimmer in favor of reduced prominence, while the thickness of both plates is the same.

In this study both designs were tested under axial and torsion loads in a material testing machine (MTS) in a composite replicate femur model, under simulated physiological loading conditions. The composite bone model provided reproducible bone properties and avoided the availability problems and variability associated with cadaver specimens. The mechanical properties of these bones have been validated [10]. The study hypothesis was that, in contrast to the open gap model test results, there is no difference in stability and stiffness between the two designs using a simulated physiologic post-operative test model for comparison.

## Methods

**Figure 3:**
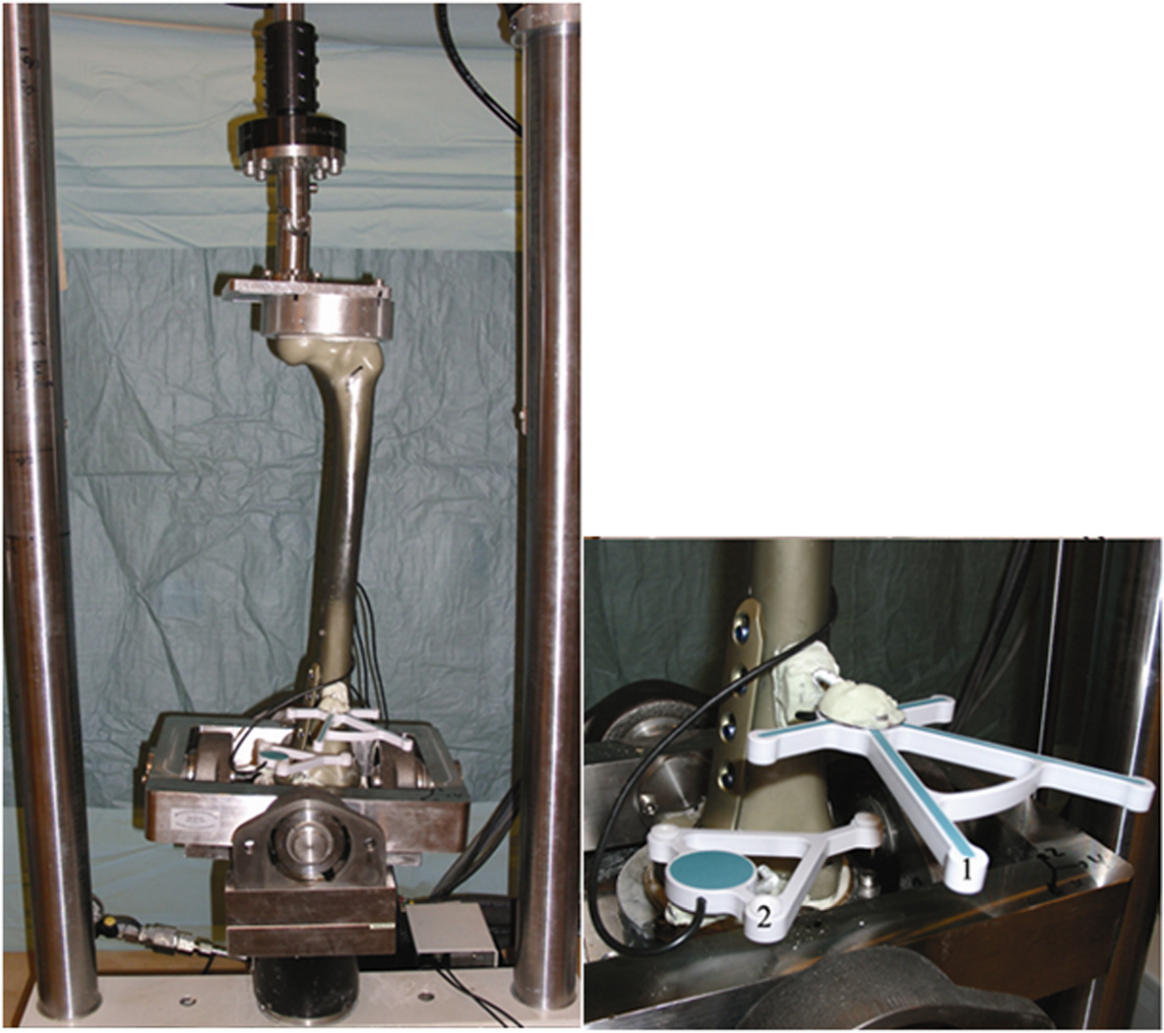
**Overview of the test setup used.** Left: the entire setup, the femur loaded in the MTS. Right: Close-up of the measuring system; 1 and 2, microphone and speaker templates.

The same test protocol as has been previously used by Brinkman et al. was used in the current study [7,8]. Fourteen femurs in total were available; 7 for each plate type (‘NEW’ , anatomical MDF plate and ‘OLD’ , antecedent MDF plate, Table 1). Bi-plane closing wedge distal femur osteotomies were performed, and the plates implanted according to standard surgical technique [11]. All femurs were aligned in a standardized way using an alignment jig and a femur saw guide (Balansys, Mathys Medical, Bettlach, Swiss), the closing wedge osteotomy was directed 20° oblique to the distal femur condylar line, a wedge of 10° was removed. The bone deformation needed for the closing of the wedge and the implant fixation were performed without producing a fracture in the opposite bone bridge. The femur head and trochanter and the distal femur end were thereafter embedded in a polyurethane-based cold-curing resin (Ureol FC 53, Vantico GmbH, Wehr, Germany) in a specially constructed fixture; the fixture allowed for mounting of the femur in a materials testing machine (MTS) (Mini Bionix, MTS Systems Corporation, Eden Prairie, MN, USA) (Figure 3). The fixture was designed in such a way that the axis of loading of the replicate femur in the MTS was through the centre of the femur head proximal and through a point 18-mm medial from the mid-condylar distance, creating a mechanical femur axis of 2°; reproducing loading in the normally aligned human knee after a supra-condylar femur osteotomy (SCO) for valgus osteoarthritis.

**Table 1:** **Overview of the configurations and test protocols**

	**Implant type**	**Axial loading**		**Torsional loading**		
		**Axial loads**		**5NM torsion load**		
				**With axial pre load**		
Total no	Test runs	1	2	3	4	5
Femurs		150 N	800 N	0 N	150 N	800 N
7	MDF OLD	100	100	100	100	100
7	MDF NEW	100	100	100	100	100
14						

Overview of the test runs: axial tests at 150 N and 800 N both 100 cycles per test run, torsion tests at 5 Nm, with 0 N, 150 N and 800 N axial pre-load, again 100 cycles per test run. ‘MDF NEW’ = anatomical MDF plate, ‘MDF OLD’ = antecedent MDF plate.

### Measuring system

The principles of rigid body motion were used to measure (micro) motion across the SCO. Reference point pairs, relative to which motion was measured, were defined on the replicate femur both proximal and distal to the osteotomy gap; two points across the midpoint of the intact cortical bone bridge, two points midway across the osteotomy and two points just posterior of the plate on the femur. Motion (displacement) of the diaphysis of the femur proximal to the osteotomy was measured relative to the femur condyles distal to the osteotomy using an ultrasound 3D motion-analysis system (CMS20S, Zebris Medizintechnik, GmbH, Isny, Germany). This system is based on the travel time measurement of ultrasonic pulses that are emitted by miniature speakers on a marker-triplet to microphones on a second marker-quartet (Figure 2). It has been previously used by Brinkman et al. in biomechanical testing of various osteotomy techniques [7,8].

### Loading protocol

The replicate femurs were subjected to axial and torsional loading protocols designed to simulate physiological loading (Table 1). Cyclical axial loading was performed at two loading levels (150 and 800 N) on all femurs, during 100 cycles for each load at a rate of 0.5 Hz. Cyclical torsional loading was thereafter performed in all femurs. Internal rotation around the Z-axis with a cyclical moment loading of 5 Nm at a rate of 0.25 Hz was applied during 100 cycles, with an increasing axial pre-load (Table 1). The different axial preloads were used to simulate no, partial and full weight bearing.

Four femurs were subsequently tested to failure under axial compression, with displacement control at a rate of 0.1 mm per second. Failure was defined by a drop of actuator loading, either because of failure of the bone, bone–implant construct, or of the implant itself. Three femurs were tested to failure under torsion, with displacement control at a rate of 0.25° per second. Criteria for failure were the same as used for the axial loading failure tests.

### Data analysis

The displacement data recorded was computed using a custom-written program in Mathematica (version 5.0, Wolfram research, Inc, Champaign, IL, USA); the change in position and the angle of rotation around all axes for each measuring point and the change in absolute distance between the measuring points was calculated. Displacement at the SCO was calculated using the change in the (absolute) distance between the measuring points per loading cycle. The amount of motion that occurs at the SCO was defined as the difference between the maximum increase and maximum decrease in the distance between measuring points; determined for each cycle and per measuring point. A greater mean difference calculated over 100 cycles and 3 measuring points indicates more motion allowed by the bone–implant construct. Stability was subsequently defined as the amount of motion allowed by the construct. A similar approach was used in the torsional tests. The amount of motion was calculated by determining the amount of rotation around the Z-axis that is allowed by the bone–implant construct during each cycle. Stability in torsion was defined as the amount of rotation allowed by the construct. The stiffness under axial compression of the construct was calculated by plotting displacement at the SCO during the failure test, defined as the average amount of movement on the Z-axis of the 3 previously defined point pairs, against the force data. Stiffness of the bone–implant construct was defined as the slope of the linear portion of the force - deformation curve (i.e. the force required per millimeter of displacement). Stiffness under torsion loading was calculated by plotting the rotation around the Z-axis over time against the moment (Nm) applied by the MTS and defined as Nm required for one degree of rotation.

### Statistical analysis

Statistical analysis was performed using SPSS statistical software (Version 11.5, SPSS, Inc, Chicago, IL, USA); the independent sample T-test was used to measure statistical differences between the motion data that was recorded and subsequently computed for the two configurations, P values < 0.05 were considered significant using a 95% confidence interval (CI95).

## Results

### Axial tests

No visible damage to bone, bone-implant construct or implant was found in any of the test runs. During each cycle of loading and unloading, a corresponding movement at the osteotomy was observed to occur. At 800 N the anatomical MDF plate allowed statistically significantly less motion than the antecedent MDF plate, at 150 N there was no difference (Table 2).

**Table 2:** **Axial and torsion test results**

			**Total runs**	**Mean**	**SD**
Axial tests	150 N	NEW plate	700	0.085651	0.054997
		OLD plate	700	0.087737	0.056193
	800 N	NEW plate	700	0.088803	0.048292
		OLD plate	700	0.096411	0.044069*
Torsion tests	0 N	NEW plate	700	0.077263	0.022999
		OLD plate	700	0.080334	0.025255*
	150 N	NEW plate	700	0.076817	0.02146
		OLD plate	700	0.078167	0.023
	800 N	NEW Plate	700	0.076932	0.021548
		OLD plate	700	0.077137	0.02275

*Statistically significantly less motion with the anatomical MDF plate design.

Results for the axial and torsion test runs, movement is in mm for the axial tests and in degrees for the torsion tests. ‘NEW plate’ = anatomical MDF plate, ‘OLD plate’ = antecedent MDF plate.

### Torsion tests

Again no visible damage to bone, bone-implant construct or implant was found in any of the test runs. In the test run with no axial preload the anatomical MDF plate allowed statistically significantly less motion than the antecedent MDF plate; in the other runs there was no difference between the two (Table 2).

### Axial failure tests

Seven failure tests (4 antecedent MDF plate, 3 anatomical MDF plate) resulted in a per-trochanteric femoral neck failure, *i.e.,* failure occurred proximally to the osteotomy in the replicate femurs. In one femur with the anatomical MDF plate a two level femur fracture occurred; a femoral neck fracture as in the other tests, but a femur shaft fracture just proximal to the plate occurred as well. No macroscopically observable failure at the bone-implant interface or of the implant itself was observed in any of the tests. No fractures of the opposing lateral cortex bone-bridge were observed with both plates. During the axial failure tests, the force time course of loading typically demonstrated increasing motion with increasing axial compression load with a sudden drop in load at failure. Calculated stiffness was similar in both plate types (Table 3).

**Table 3:** **Axial and torsion failure test results**

**Femur**	**NEW axial**	**OLD**	**NEW Torsion**	**OLD**
1	4313.10	4240.50	31.00*	32.10*
2	4242.90	4193.50	33.40	37.20
3	4011.20	5002.10	29.50	26.50
4	3607.60	4084.80		
Average	4043.70	4380.23	31.30	31.93
Significance	P = 0.27		P = 0.78	
(p < 0.05 = sign)				

Results for the axial and torsion failure tests, axial force is in N/mm, torsion force is Nm/°; differences are not statistically significant.

*Femurs in which the drive-shaft of the MTS came loose.

‘NEW’ = anatomical MDF plate, ‘OLD’ = antecedent MDF plate.

### Torsion failure tests

In the 6 femurs that were tested to failure under torsion loads, different patterns of failure were observed. Two femurs (one anatomical MDF plate, one antecedent MDF plate) did not fail, the test ended because the drive-shaft of the MTS itself came loose, which became apparent by a sudden drop in load on the femur. In those two there was no visible damage to the construct, their calculated stiffness did not differ from the other femurs. In two (again one anatomical MDF plate, one antecedent MDF plate) the test ended because of a fracture at the biplane hinge on the lateral cortex bone-bridge. And in two (one anatomical MDF plate, one antecedent MDF plate) a spiral fracture occurred just proximal of the osteotomy, just anterior of the proximal screw-bone interface. Calculated stiffness again was similar in both plates (Table 3).

## Discussion

The most important finding in this study was that in contrast to the results of the open gap tests, the anatomical MDF plate was not found to be statistically significantly less stable and stiff than the antecedent MDF plate. In two test runs the anatomical MDF plate allowed statistically significantly less motion than the antecedent MDF plate (800 N axial test and the torsion test with no axial pre-load). In all the other tests there was no statistically significant difference between the two. Again as in previous studies the axial failure tests ended because of per-trochanteric femoral neck fractures, not because of failure at the osteotomy^7^. Stiffness in the current tests also was very similar in both plates, with the antecedent MDF plate on average showing a slightly higher stiffness, but not statistically significant. In the torsion failure tests, the failure modes differed somewhat between femurs, but again there was no difference between the two plates. Again the antecedent MDF plate showed a slightly higher stiffness on average, but not statistically significant.

In these tests, as in previous tests by Brinkman et al., the post-operative situation after medial closing wedge SCO was reproduced as “life-like” as possible, with simulated partial and full weight bearing and simulated leg alignment as in patients that undergo SCO [7,8].

In the open gap model which is often used to compare the mechanical strength of osteosynthesis implants, a composite cylinder is cut in half and both ends are fixated with a plate (Figure 1A). That construct is then tested under axial and torsion loads, with motion measured across the gap. As documented by Stoffel et al. gap size and plate length are important factors in the overall stability [2]. It is therefore not unexpected that under these circumstances a shorter and slimmer plate has a lower stiffness and fatigue strength than a plate that is longer and that has an expanded shaft.

Various authors have compared different types of plates and nails, and different types of screw configurations, using sawbones or human cadaver bones, in distal femur and proximal tibia fracture models [1,3–6,9]. Almost all use some form of gap model; with the gap representing the unstable fracture [1,3–6]. David et al. used anatomic reduction without fracture gaps in a cadaver study testing a locked nail and an angled blade plate [9]. They stated that direct bony opposition more closely approximates the clinical situation, in which fracture fragments are reduced anatomically before placement of the fixation device. So as a consequence of the fracture reduction in most fractures stability is likely to be increased, which is not simulated in gap models. Similarly in closing wedge SCO there is no gap, both ends of the osteotomy are rigidly compressed against each other. And because of the controlled nature in which the SCO is created, closed and fixated there is inherent stability of the construct. Furthermore because of the gap in gap model testing the loading conditions at the plate are completely different, in a gap model the plate is loaded under tension, in the current setup under compression, because it is on the medial side of the femur. In SCO the load at the knee is shifted from lateral to medial, therefore after SCO the compressive forces will be on the medial side, as is the case in the current test setup.

The above arguments can also be made against concerns over the inferior results of the anatomical MDF plate in the fatigue tests using an open gap test model; they were not performed under simulated post-operative conditions. Fatigue testing with an open gap model may give a better understanding of the plate’s failure mode. However it is questionable whether fatigue test results are representative of the clinical situation in SCO, because ongoing bone-healing will stabilize the construct as the implant fatigues, i.e., load sharing takes place decreasing the loading on the plate. Closing wedge SCO typically heal in 6 – 12 weeks, specific postoperative rehabilitation protocols provide for partial and full weight-bearing tailored to stability of fixation construct and time to full bone healing [12]. In our experience bone healing using a bi-plane osteotomy technique is fast and increased stability through bone healing occurs quickly [11]. This whole process is not simulated in any biomechanical test setup and can only be documented in in-vivo bone healing studies.

Limitations of the study are that, because of the small number of femurs available, the failure data set is small in size. No fatigue tests were performed, due to data size collection and time limitations. The MTS is not able to reliably load the construct quickly therefore fatigue testing of a single femur would have taken weeks. No power calculations were performed and no test- re-test reliability measurements were performed.

As with all experimental tests, the test setup used does not account for the variation in host biology and is a representation of what could happen in vivo and is for sure a simplification. The forces that are exerted on a leg during flexion and extension were not simulated; simple axial and torsional loading was used. These limitations are true however for most if not all types of biomechanical testing models. Furthermore the data is not comparable to the two previously published biomechanical studies by Brinkman et al., because 4^th^ generation sawbones were used instead of 3^rd^ as the 3^rd^ generation sawbones are no longer commercially available [7,8]. According to Heiner the 4^th^ generation bones mirror human bones more closely than the 3^rd^ [10]. Tests with the 4^th^ generation bones should therefore, if anything, be more representative of the post-operative situation after SCO.

## Conclusions

The current test results show that under simulated physiological loading conditions there is no difference in stability between the two plates; the latest design Tomofix MDF provides as much, if not more, stability and equal stiffness compared to the antecedent design. These test results stress the importance of not only using standard open gap and 4-point-bending tests, but also to use as life-like as possible test conditions for any form of biomechanical testing of new implants.
